# The dynamics of protein body formation in developing wheat grain

**DOI:** 10.1111/pbi.12549

**Published:** 2016-03-15

**Authors:** Katie L. Moore, Paola Tosi, Richard Palmer, Malcolm J. Hawkesford, Chris R.M Grovenor, Peter R. Shewry

**Affiliations:** ^1^ School of MaterialsUniversity of ManchesterManchesterUK; ^2^ School of Agriculture Policy and DevelopmentReading UniversityReadingUK; ^3^ Rothamsted ResearchHarpendenUK; ^4^ Department of MaterialsUniversity of OxfordOxfordUK

**Keywords:** endosperm development, NanoSIMS, protein deposition, wheat

## Abstract

Wheat is a major source of protein in the diets of humans and livestock but we know little about the mechanisms that determine the patterns of protein synthesis in the developing endosperm. We have used a combination of enrichment with ^15^N glutamine and NanoSIMS imaging to establish that the substrate required for protein synthesis is transported radially from its point of entrance in the endosperm cavity across the starchy endosperm tissues, before becoming concentrated in the cells immediately below the aleurone layer. This transport occurs continuously during grain development but may be slower in the later stages. Although older starchy endosperm cells tend to contain larger protein deposits formed by the fusion of small protein bodies, small highly enriched protein bodies may also be present in the same cells. This shows a continuous process of protein body initiation, in both older and younger starchy endosperm cells and in all regions of the tissue. Immunolabeling with specific antibodies shows that the patterns of enrichment are not related to the contents of gluten proteins in the protein bodies. In addition to providing new information on the dynamics of protein deposition, the study demonstrates the wider utility of NanoSIMS and isotope labelling for studying complex developmental processes in plant tissues.

## Introduction

Wheat is one of the three major cereal crops which feed the human race and the major staple crop in temperate countries. Because of this, it is a major source of protein for human health. For example, bread alone provides over 10% of the daily protein intake in UK adults (Steer *et al*., 2008). This contribution is more important in low‐income groups and particularly important in some developing countries where wheat provides between 50 and 70% of the total calories (Cakmak, 2002). Despite this, we know little about the mechanisms that determine protein content and deposition and hence lack the tools required to improve this aspect of grain quality.

The major storage tissue in the cereal grain is the starchy endosperm, which comprises about 80% starch and about 10% protein. This is a highly organized and differentiated tissue, with significant gradients in cell composition, particularly protein that is concentrated in the outer few layers of starchy endosperm cells (called subaleurone cells) in all cereals, including wheat (Tosi *et al*., 2011) and rice (Ohdaira *et al*., 2011). These gradients will affect the recovery of protein in fractions produced by grain processing, such as milling of wheat to give white flour and polishing of rice. Both of these processes can result in significant loss of protein from the human diet if the outer layers of the starchy endosperm are removed with the bran.

Little is known about when the protein gradients in the starchy endosperm are established, or the mechanisms that determine them. However, it is known that the subaleurone cells of the cereal endosperm have a different origin from the central endosperm cells, being derived from the aleurone cells which retain their ability to divide both anticlinally and periclinally up to about 12 days postanthesis (dpa). The inner cells from these divisions redifferentiate into subaleurone cells with only a single layer of aleurone cells being present in the mature wheat grain (Becraft and Yi, 2011). Hence, the accumulation of protein could be associated with the redifferentiation of the subaleurone cells into a new type of protein‐rich storage tissue.

Secondary ion mass spectrometry (SIMS) has a number of advantages over conventional methods for imaging minerals in plant tissues, including high sensitivity (parts per million) and the ability to discriminate between isotopic forms of elements (Wilson *et al*., 1989). It has been applied previously to plant tissues (Grignon *et al*., 1997; Jauneau *et al*., 1994), including determining cereal grain composition (Feeney *et al*., 2003; Heard *et al*., 2002; Moore *et al*., 2010, 2012b). However, these studies were all of static systems, whereas we show here that SIMS can also be combined with isotopic labelling to study complex dynamic processes in developing tissues. We have therefore combined the *in vivo* incorporation of ^15^N‐labelled glutamine, the main transported form of nitrogen in wheat (Fisher and Macnicol, 1986), into developing caryopses with NanoSIMS analysis of tissue sections. The high lateral resolution of the NanoSIMS allowed the precise ratio of ^15^N:^14^N to be determined in individual protein bodies, providing the first description of the dynamics of nitrogen transport in relation to protein deposition.

## Results

Developing wheat grains (caryopses) were fed ^15^N‐glutamine via the rachis (Figure S1) at two stages of development: at 10 dpa, corresponding to the start of grain filling, and at 20 dpa, corresponding to the middle of the grain‐filling period. This treatment did not affect grain development, and caryopses were removed and analysed for ^15^N enrichment at 6 h, 24 h and 7 days after the start of application. Analyses of whole caryopses confirmed that there was substantial enrichment with ^15^N, which accounted for over 8% of the total N present at 6 h from the beginning of the feeding period at 10 dpa (when over 1 ml of the total 1.8 ml of ^15^N supplied to each ear had been taken up). Enrichment remained high (over 10%) at 24 h (at 11 dpa) after the beginning of the feeding period but fell to about 5% after 7 days (at 17 dpa) due to dilution with ^14^N from the normal uptake and transport of nitrogen from the soil. By contrast, transport from the rachis to the spike and into the caryopses was slower when the ^15^N was fed at 20 dpa, enrichment being only about 1.8% after 6 h (when about 400 μl of solution had been taken up) and increasing to about 5% after 24 h. Enrichment decreased slightly, to about 4%, after 7 days, presumably due again to dilution with ^14^N taken up from the soil (Table S1).

**Figure 1:**
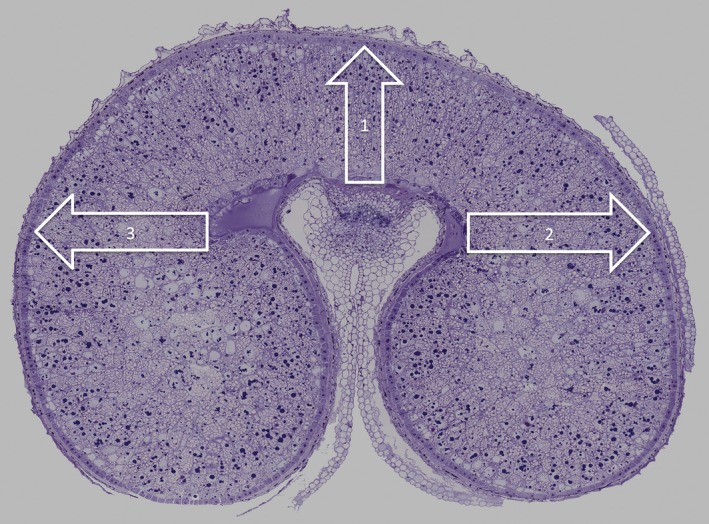
Transverse section of developing grain of wheat at 17 days postanthesis (dpa) stained with toluidine blue and annotated to show the positions and directions of the three transects selected for NanoSIMS analysis.

To study the incorporation of ^15^N into proteins in the tissues, rather than into amino acids and other low‐molecular‐weight compounds, sections of developing caryopses from these times were fixed using paraformaldehyde and glutaraldehyde. This type of preservation, known as chemical fixation, adequately preserves elements that are covalently bound in large molecules, such as proteins, but will wash out diffusible ions, such as K and Na, and free amino acids that were not of interest in this study (Moore *et al*., 2012a). Transverse sections of the developing caryopses were prepared by ultramicrotomy and stained with toluidine blue to show protein distribution in the starchy endosperm (Figures 1 and S2). Serial sections, adjacent to those used for light microscopy, were prepared for NanoSIMS analysis to determine the patterns of protein enrichment.

NanoSIMS analyses showed little ^15^N enrichment in either the maternal or endosperm tissues after 6 h feeding at either 10 or 20 dpa, although mass spectroscopy of the whole (un‐fixed) caryopses showed 8% and 1.8% enrichment, respectively. Free glutamine would not have been preserved during sample preparation for microscopy, and it was therefore concluded that little or none of the ^15^N transported to the endosperm had become incorporated into proteins at this time. The 6‐hour samples were therefore not studied further. By contrast, clear enrichment of endosperm proteins with ^15^N was observed at 24 h and 7 days after feeding at both stages of development. Three transects of cells across the starchy endosperm were therefore selected for detailed study: one extending from the nucellar projection to the dorsal surface of the grain (transect 1) and two extending laterally across the lobes from the nucellar projection (transects 2 and 3) (Figure 1). Essentially similar results were obtained for these three transects so only data for transect 1 are reported here (see Figure S3 for a comparison of the three transects at three different time points).

**Figure 2:**
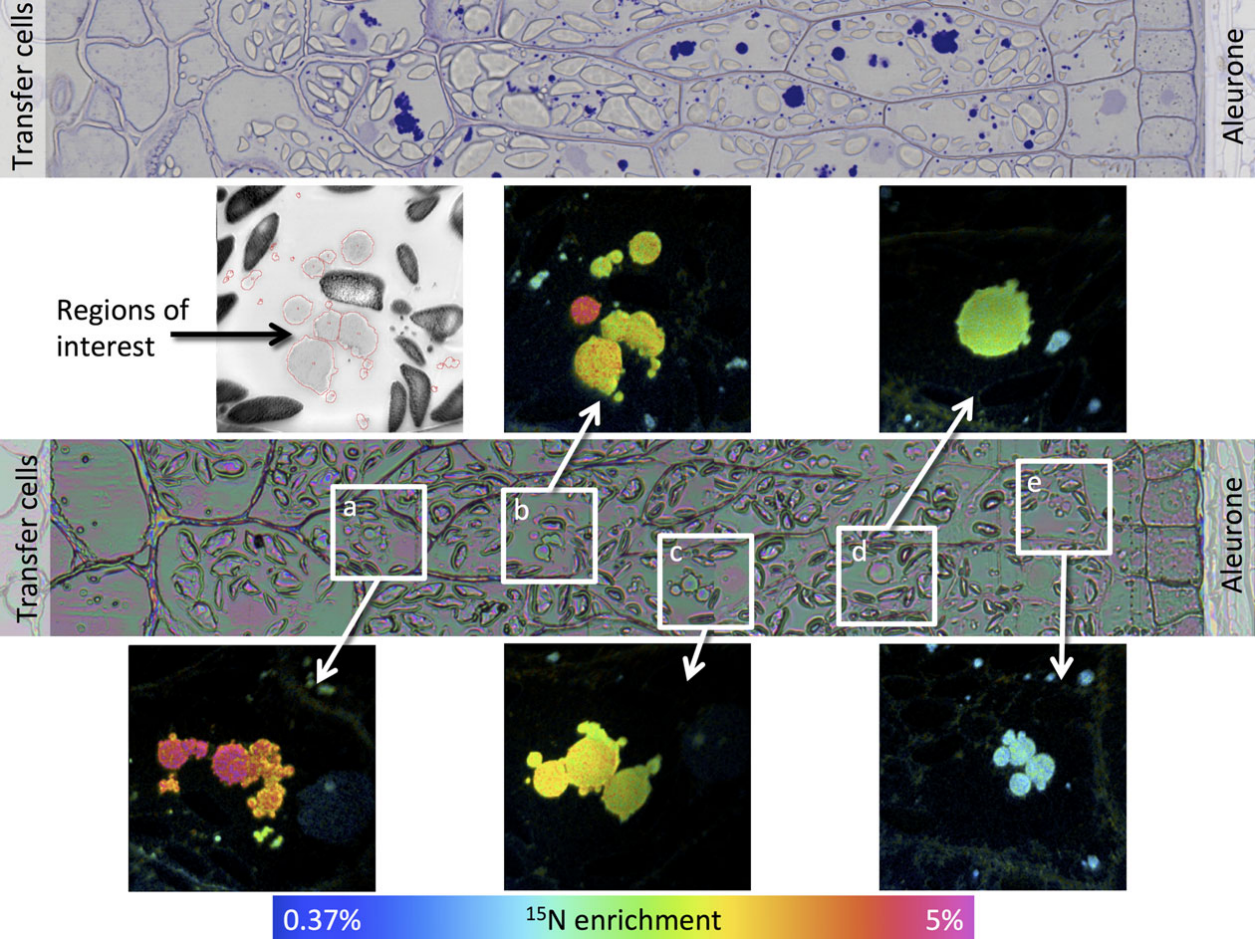
Analysis of transect 1 of a wheat starchy endosperm taken from a developing caryopsis at 17 dpa, after feeding ^15^N glutamine at 10 dpa. The top panel shows a conventional light microscopy image after staining with toluidine blue to identify protein bodies. The central panel shows an optical image from a serial section which was used to select the areas where secondary ion NanoSIMS images were acquired at high lateral resolution. Areas a to e marked on this optical image are expanded in the boxes, and enrichment with ^15^N is shown using a hue saturation intensity colour scale with the ^15^N enrichment shown in the scale at the bottom. The grey scale image is a NanoSIMS secondary electron image from area b. Regions of interest, protein deposits, are outlined in red and indicate the regions from which the ^15^N enrichment data were extracted.

**Figure 3:**
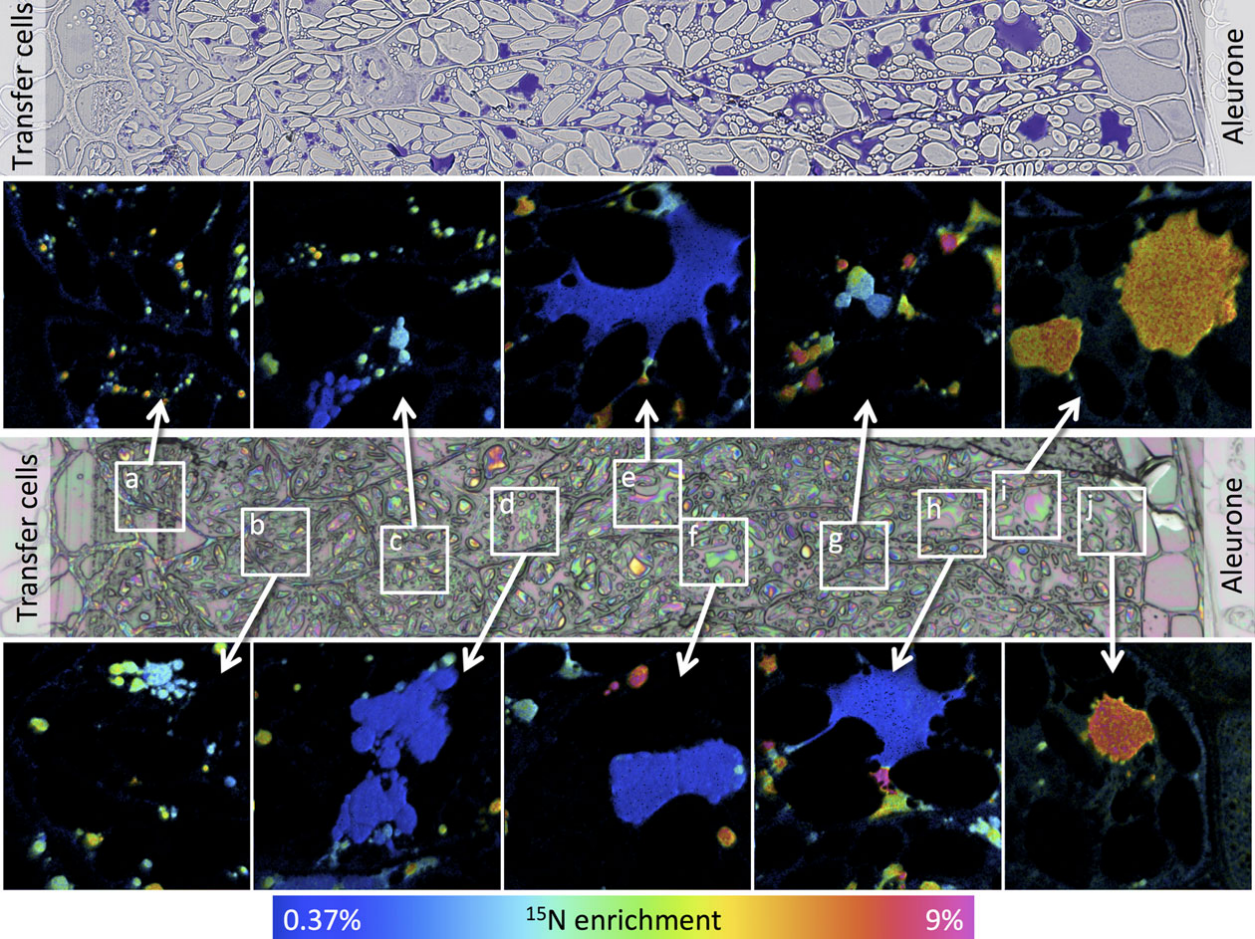
Analysis of transect 1 of a wheat starchy endosperm taken from a developing caryopsis at 27 dpa, after feeding ^15^N glutamine at 20 dpa. The top panel shows a conventional light microscopy image after staining with toluidine blue to identify protein bodies. The central panel shows an optical image from a serial section which was used to select the areas where secondary ion NanoSIMS images were acquired at high lateral resolution. Areas a to j marked on this optical image are expanded in the boxes and enrichment with ^15^N is shown using a hue saturation intensity colour scale with the ^15^N enrichment shown in the scale at the bottom.

Data for caryopses at 7 days after the start of feeding with ^15^N glutamine are presented in Figure 2 (17 dpa) and Figure 3 (27 dpa) and data for 24 h after feeding (11 and 21 days) in Figures S4 and S5, respectively. The upper panel of Figure 2 shows a transect from a serial microtome section close to the NanoSIMS section, taken from a caryopsis at 17 dpa (7 days after feeding at 10 dpa) stained with toluidine blue to reveal protein. This shows the presence of small protein bodies deposited within vacuoles in all cell layers, from close to the transfer cells (area a) to the subaleurone cells (area e). A similar transect taken at 27 dpa (7 days after feeding at 20 dpa) (Figure 3, upper panel) reveals more extensive protein deposits, which vary in size and include both small newly initiated bodies and the fusion of protein bodies to give large irregular deposits.

The lower panels of Figures 2 and 3 show optical images which were used to select the areas where secondary ion NanoSIMS images have been acquired at high lateral resolution (areas a‐e in Figure 2, a‐j in Figure 3). The expanded images of these areas are shown as hue saturation intensity colour maps of the ^15^N:^14^N ratio with the level of ^15^N enrichment being indicated by the colour scale at the bottom of each of figure. The NanoSIMS analysis shows clear differences in the extent of enrichment across the transects and between individual protein bodies within single cells. For example, Figure 2 shows greater enrichment of the protein bodies in the starchy endosperm cells closest to the transfer cells (i.e. close to the point of entry of the ^15^N label, areas a and b), while Figure 3 shows high enrichment of large protein bodies in the outer endosperm cells (farthest from the transfer cells, areas i and j) but also of small protein bodies in the cells closest to the transfer cells (areas a and b).

**Figure 4:**
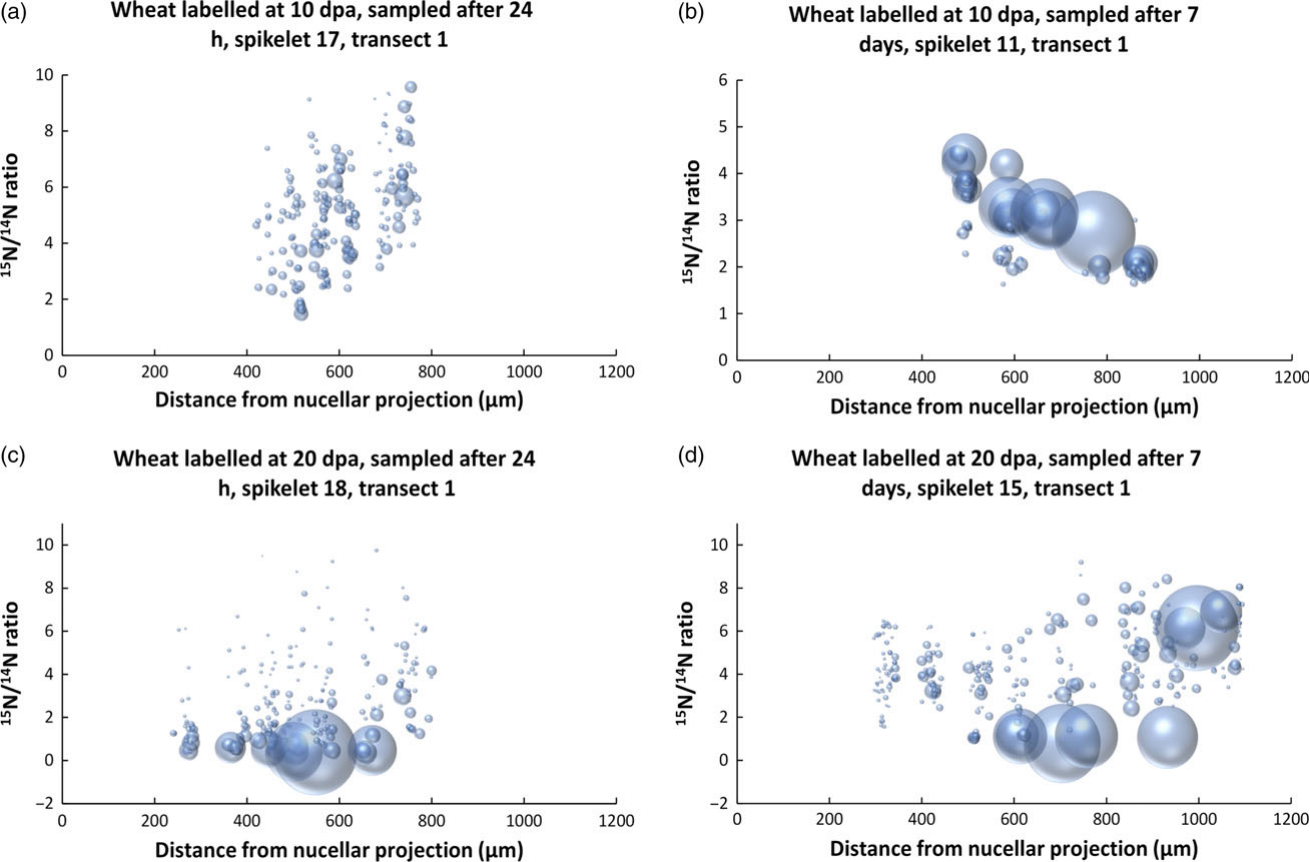
Graphical representation of the size and ^15^N enrichment of protein bodies along transect 1 of starchy endosperm tissue after labelling at 10 dpa 24 h (a), 10 dpa 7 days (b) 20 dpa 24 h (c) and 20 dpa 7 days (d). Individual protein bodies are displayed as ‘bubbles’, which correspond in size to their measured areas. The positions of the protein bodies correspond to their locations along the transect (*x*‐axis) and their degree of enrichment with ^15^N (*y*‐axis).

It is clearly not possible to make a quantitative comparison of enrichment patterns by visual inspection alone. Transects from caryopses of each time stage (24 h and 7 days after labelling at 10 and 20 dpa) were therefore analysed in detail to measure the areas and enrichment levels of individual protein bodies, with over 6000 individual protein deposits being analysed in total. Examples of this analysis are displayed graphically in Figure 4, with individual protein bodies displayed as ‘bubbles’, the sizes of which correspond to their measured areas. The positions of the ‘bubbles’ along the *x*‐axis corresponds to their location along the transect, while their degree of enrichment with ^15^N is shown on the *y*‐axis. Replicate analyses from different spikelets (Figure S6) show a high level of similarity.

The increase in the distance of the outermost protein bodies from the nucellar projection (see Figures 4, S3 and S6) is due to expansion of the endosperm during the period from 10 to 27 dpa, which corresponds under our controlled environment growth conditions to the point of maximum grain size, and may be due to cell division, cell expansion or a combination of these. In fact, counts of cells along transect 1 showed a small decrease in the number of endosperm cells after 10 dpa: from 11.7 + /− 1.2 at 11 dpa to 10.9 + /− 1.3 at 17 dpa, 10.8 + /− 1.5 at 21 dpa and 10.8 + /− 0.8 at 27 dpa. This cell loss is consistent with previous studies (Gao *et al*., 1992) and occurs despite continued anticlinal division of the aleurone cells until at least 14 dpa (Bechtel and Wilson, 2003).

The graphs demonstrate that the protein bodies present at 11 dpa were numerous but small, with enrichment increasing towards the outside of the grain at 24 h after labelling (Figure 4 a). At 17 days, the number of protein bodies had decreased and their average size increased massively, presumably due to fusion. However, small (presumably newly initiated) protein bodies were present in the same cells (Figure 4 b). The level of enrichment also decreased from the inner to outer cells at 17 days, which was probably due to dilution of the protein in the outer cells with ^14^N. These results therefore show that the ^15^N was rapidly transported into the caryopses and incorporated into small protein bodies (by 24 h) which subsequently grew in size, particularly in the outer layers of the starchy endosperm.

Analysis of the 21 dpa samples (i.e. at 24 h after labelling) showed large and small protein bodies, with the smaller bodies being more highly enriched in ^15^N (Figure 4 c), indicating that they had been recently initiated. Differences were observed at 27 days (7 days after labelling). Firstly, whereas the central cells contained small highly enriched protein bodies, a similar level of enrichment was observed in the large protein bodies in the outer (most recently formed) subaleurone cells (Figure 4 d). This pattern may result from a decreased level of protein synthesis and deposition in the central cells combined with a continued high level of protein synthesis in the protein‐rich subaleurone cells.

Differences in protein body morphology were also observed between the inner and outer starchy endosperm cells, particularly at 27 dpa (Figure 3). The large protein bodies in the inner cells appeared to be formed by the fusion of small protein bodies (Figure 3 panels a‐h) and were irregular in shape (Figure 3 e, f, h), probably because they became squashed between expanding starch granules. By contrast, those in the subaleurone cells (which contained fewer starch granules) were more regular in shape and appeared to grow by direct accumulation of newly synthesized protein as well as fusion (Figure 4 i and j).

**Figure 5:**
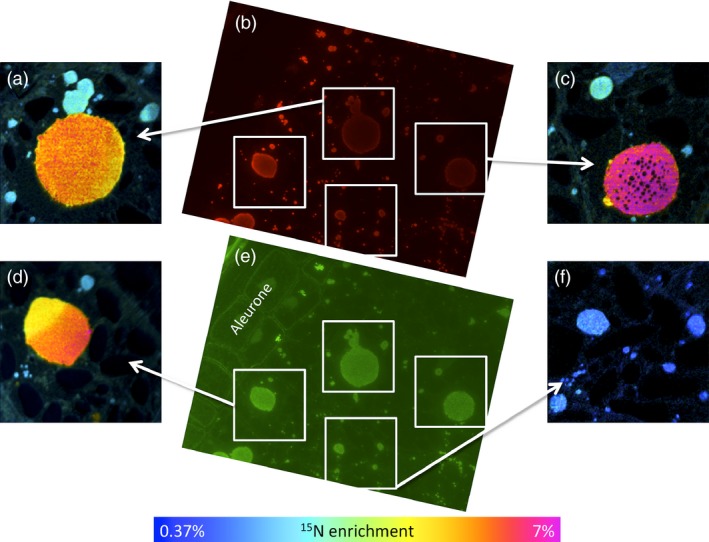
Comparison of ^15^N enrichment of protein bodies in starchy endosperm cells at 17 dpa (7 days after labelling at 10 dpa) determined by NanoSIMS (panels a, c, d, f) with the contents of gluten proteins determined by immunolabeling with the monoclonal antibody IFR0610 which recognizes gliadins and low‐molecular‐weight subunits (red, panel b) and the polyclonal antibody R2‐HMG which recognizes high‐molecular‐weight subunits of glutenin (green, panel e). The primary antibody binding in panel b was detected using an Alexa 568 anti‐mouse conjugated antibody, while in panel e, an Alexa 488 anti‐rabbit conjugated antibody was used. In panels a, c, d and f, enrichment with ^15^N determined by NanoSIMS is displayed using a hue saturation intensity colour scale. Comparison of immunofluorescence and NanoSIMS images suggests that ^15^N incorporation is independent of protein body composition, reflecting instead time of synthesis in relation to ^15^N labelling.

The major protein components stored in the starchy endosperm cells of wheat are prolamin storage proteins, which correspond to the gluten proteins that underpin the processing properties of wheat flour and dough. Gluten comprises over 50 individual components, which are divided broadly into polymeric glutenins and monomeric gliadins. These two protein groups differ little in their timing of synthesis during grain development (Shewry *et al*., 2009), but it has been suggested that they are partially segregated into different types of protein body. In particular, the gliadins appear to be enriched in protein bodies of vacuolar origin while glutenins appear to accumulate directly within the lumen of the endoplasmic reticulum (Rubin *et al*., 1992; Tosi *et al*., 2009). It was not possible to discriminate between these two types of protein body in this study, although it is clear that the large aggregated structures present at later stages of development represent aggregates of small protein bodies within vacuoles. However, we did determine whether the differences in enrichment of protein bodies within cells was related to their protein composition by analysing serial sections by NanoSIMS and by immunomicroscopy using specific antibodies for two major classes of gluten protein (Figure 5). The monoclonal antibody IFR0610 (red, Figure 5 b) recognizes gliadins and low‐molecular‐weight subunits (Brett *et al*., 1999), which are classified as sulphur‐rich prolamins, while the polyclonal antibody R2‐HMW (green, Figure 5 e) recognizes epitopes on high‐molecular‐weight subunits of glutenin (Mills *et al*., 2000). Comparison of the labelling patterns with these two antibodies showed no relationship between protein composition and the degree of enrichment with ^15^N (Figure 5).

## Discussion

We have demonstrated that a combination of isotopic labelling and NanoSIMS imaging can be used to determine the spatial and temporal dynamics of protein deposition in the developing wheat endosperm. However, the interpretation is not straightforward as transport and deposition are accompanied by dilution with ^14^N.

Firstly, we have demonstrated that ^15^N provided as glutamine is transported radially across the developing starchy endosperm, from the transfer cells to the subaleurone cells. However, the rate of transport appears to be slower as the endosperm develops, with enrichment of the subaleurone cells being observed within 24 h when the labelling was carried out at 10 dpa, but not at 20 dpa. Clear differences in ^15^N enrichment were also observed at 7 days after labelling compared with 24 h after labelling, for both developmental stages. Dilution with ^14^N may account for the failure to observe enrichment of the subaleurone cells at 17 days, whereas the enrichment observed at 27 dpa may reflect lower dilution combined with a slower rate of ^15^N transport.

The studies described here do not provide information on the mechanisms that determine the patterns of protein accumulation but it can be speculated that the subaleurone cells, which have a different and more recent origin than the central starchy endosperm cells (originating from divisions in the aleurone cells late in development), have a higher requirement for amino acids as they are programmed to store high levels of protein (up to 40% dry weight). This strong sink activity may result in a gradient which effectively drives amino acid transport from the transfer cells across the central starchy endosperm to the subaleurone cells, and this may explain why the large protein bodies in the subaleurone cells are highly enriched at 27 days.

Finally, we have shown that the larger protein deposits in older starchy endosperm cells are formed by the fusion of small protein bodies and that small protein bodies can also be observed in these same cells. Furthermore, some of these small protein bodies were highly enriched with ^15^N at 21 and 27 dpa, indicating a continuous process of protein body initiation, in both older and younger starchy endosperm cells and in all regions of the tissue. This contrasts with the deposition of starch which shows two clear phases of granule initiation, with large A granules being initiated up to 5 dpa and small B granules between 9 and 14 dpa (Stone and Morell, 2009).

Hence, the analyses reported here clearly show that the nitrogen required for the synthesis of storage proteins in the wheat starchy endosperm is transported radially across the tissue from the groove and transfer cells, rather than around the circumference. Secondly, although a gradient in protein concentration is established, the initiation and expansion of protein bodies occur continuously in all cells, irrespective of their ontogeny or position.

This novel information has wider relevance to developmental processes in other cereals and is important if we wish to manipulate the pattern of protein accumulation to improve grain quality. It also demonstrates that combination of NanoSIMS and isotope labelling has potential for wider applications for analysing dynamic processes in other complex biological systems, such as seeds and lignified tissues in which the more widely used confocal imaging cannot be applied due to the opacity of the material.

## Experimental procedures

### Plant material and application of ^15^N glutamine

Plants of bread wheat cv Cadenza were grown in controlled environment rooms at Rothamsted Research at 18**°**C day/15**°**C night temperature with a photoperiod of 16 h provided by banks of 400W hydrargyrum quartz iodide (HQI) lamps (Osram Ltd, UK) generating a light intensity of ~700 μmol/m^2^/s photosynthetically active radiation (PAR) at the pot surface.

Ears at 10 dpa and 20 dpa were fed 1.6 ml of a 34 mM ^15^N glutamine (L‐Glutamine‐(*amine*‐^15^N) 98 atom % ^15^N, Sigma‐Aldrich) solution via a glass capillary tube (Drummund Microcaps, Sigma‐Aldrich) inserted into the rachis through a small incision between the first two basal spikelets, and immersed in an Eppendorf tube containing 1.8 ml of the labelled amino acid solution. Uptake of the solution by capillarity proceeded at different rates in different plants and was generally faster in ears at 10 dpa than at 20 dpa. To ensure that a minimum of 1.6 ml of ^15^N‐labelled glutamine solution was taken up by the ears within a 24‐hour period, capillary tubes were regularly checked for blockages and re‐inserted if necessary. The feasibility and efficiency of such a capillary feeding method was previously tested using a solution of aniline blue dye (0.5% w/v) (Figure S1). Dissection and analysis of the ears showed that the solution had reached all of the grains in all the spikelets after 4 h from the start of feeding. Because the feeding was carried out on intact plants, the spikes and caryopses developed normally and were harvested immediately after the 6‐hour feeding period and then after 24 h and 7 days. Different ears were used for each labelling treatment and collection time point with caryopses from the 10‐11th and 16‐17th spikelets being used for analysis.

### Preparation of grain sections

Transverse sections (approximately 1 mm thick) were cut in fixative (2.5% (w/v) paraformaldehyde, 0.5% (w/v) glutaraldehyde in 0.1M Sorenson's phosphate buffer, pH 7.2) from the middle of the grain. Sections were fixed at room temperature for 4 h and then rinsed three times in buffer before dehydration in an ethanol series immediately followed by infiltration with LR White resin (medium grade, TAAB L012) for several days. Resin‐infiltrated samples were polymerized in polyethylene capsules (TAAB) at 55°C.

The embedded wheat grains were sectioned using a Reichert‐Jung Ultracut ultramicrotome to a thickness of 1 micron, collected on drops of distilled water on silicon wafers and dried on a hot plate at 40 °C to stretch them flat. To prevent charging during NanoSIMS analysis, the samples and substrates were coated with 10 nm of platinum before loading into the NanoSIMS.

### NanoSIMS

SIMS analysis was carried out with a NanoSIMS 50 instrument. A 16 keV Cs^+^ beam with a current of 1.2‐3.2 pA was scanned over the sample surface, and the bombardment resulted in the generation of negative secondary ions. These secondary ions were analysed by mass using a double focusing mass spectrometer. Detectors were aligned to detect ^12^C^−^, ^16^O^−^, ^12^C^14^N^−^, ^12^C^15^N^−^ and ^31^P^−^ with most detectors aligned using the sample except for the ^31^P^−^ which was aligned using a standard of GaP. To make quantitative comparisons between different samples and ensure that the analysed area was at steady state, Cs^+^ ions were implanted into the surface to achieve a dose of 1 × 10^17^ ions cm^−2^. Measurements of a control sample were acquired with an image dwell time of 20 ms, while images of the ^15^N‐labelled samples were acquired with a dwell time of 30 ms and at an image size of 50 ×× 50 μm and 256 by 256 pixels. Image J with the OpenMIMS plugin (Harvard, Cambridge, MA, USA) was used to generate isotope ratio images and extract quantitative data. Hue saturation intensity (HIS) colour maps were used to display the isotopic variation, the colour scale being set so that blue represented the natural background level of ^15^N (0.37%) and pink the highest ratio found in the set of images from each transect.

For each of the four different time stages, two sections were examined taken from duplicate grains from the same ears. Three transects were taken from each section as shown in Figure 1. In total, 6025 individual protein bodies were measured for size and enrichment.

### Light and immunomicroscopy

Semi‐thin sections were cut using a Reichert‐Jung Ultracut ultramicrotome, collected on drops of distilled water on multiwell slides coated with poly‐L‐lysine hydrobromide (Sigma) and dried on a hot plate at 40°C. Sections were stained with 0.01% (w/v) toluidine blue in 1% (w/v) sodium tetraborate, pH9. Immunofluorescence analysis was carried out as described in Tosi *et al*. (2011), using the antibodies R2–HMG rabbit polyclonal (specific for high‐molecular‐weight (HMW) glutenin subunits) and IFRN 0610 mouse monoclonal (recognizing epitopes present on gliadins and low‐molecular‐weight (LMW) glutenin subunits).

### Bulk ^15^N enrichment analysis by mass spectroscopy

Hand‐dissected caryopses were frozen in liquid nitrogen and reduced to powder using a mortar and pestle. A known volume of 0.5 mg samples (± 0.1 mg) were analysed for total %N with a Vario Micro Elemental Analyser (Elementar Analysis Systems, Hanau, Germany) connected to an Isoprime 100 isotope ratio mass spectrometer (Isoprime Ltd, Cheadle Hume, UK) to determine ^15^N enrichment, using commercial wheat flour as a control for the natural baseline level of ^15^N.

## Supporting information


**Figure S1** Uptake of aniline blue by a wheat ear at 10 dpa via capillary tube feeding.
**Figure S2** Transverse sections of the developing caryopses and stained with toluidine blue.
**Figure S3** A graphical comparison of the three transects shown in Figure 1 showing the size and enrichment of the protein bodies.
**Figure S4** Analysis of transect 1 of a wheat starchy endosperm taken from developing caryopses at 11 dpa, after feeding ^15^N at 10 dpa.
**Figure S5** Analysis of transect 1 of a wheat starchy endosperm taken from developing caryopses at 21 dpa, after feeding ^15^N at 20 dpa.
**Figure S6** A comparison of the transects taken from grains labelled at 10dpa 24 h, 10 dpa 7 days, 20 dpa 24 h and 20dpa 7 days.
**Table S1** Bulk enrichment data from developing wheat caryopses at 6 h, 24 h and 7 days.
