# Supplementary material for: The dynamics of protein body formation in developing wheat grain
Source: Plant Biotechnol J. 2016 Mar 15;14(9):1876–82. doi: 10.1111/pbi.12549 (PMC4988504; doi:10.1111/pbi.12549)
Supplement: Supplementary file 1 — Figure S1 Uptake of aniline blue by a wheat ear at 10 dpa via capillary tube feeding. Figure S2 Transverse sections of the developing caryopses and stained with toluidine blue. Figure S3 A graphical comparison of the three transects shown in Figure 1 showing the size and enrichment of the protein bodies. Figure S4 Analysis of transect 1 of a wheat starchy endosperm taken from developing caryopses at 11 dpa, after feeding 15N at 10 dpa. Figure S5 Analysis of transect 1 of a wheat starchy endosperm taken from developing caryopses at 21 dpa, after feeding 15N at 20 dpa. Figure S6 A comparison of the transects taken from grains labelled at 10dpa 24 h, 10 dpa 7 days, 20 dpa 24 h and 20dpa 7 days. Table S1 Bulk enrichment data from developing wheat caryopses at 6 h, 24 h and 7 days. [file PBI-14-1876-s001.pdf]

Supporting Information

The Dynamics of Protein Body Formation in Developing Wheat Grain

Authors: Katie L Moore, Paola Tosi, Richard Palmer, Malcolm J Hawkesford, Chris R M Grovenor and Peter R Shewry

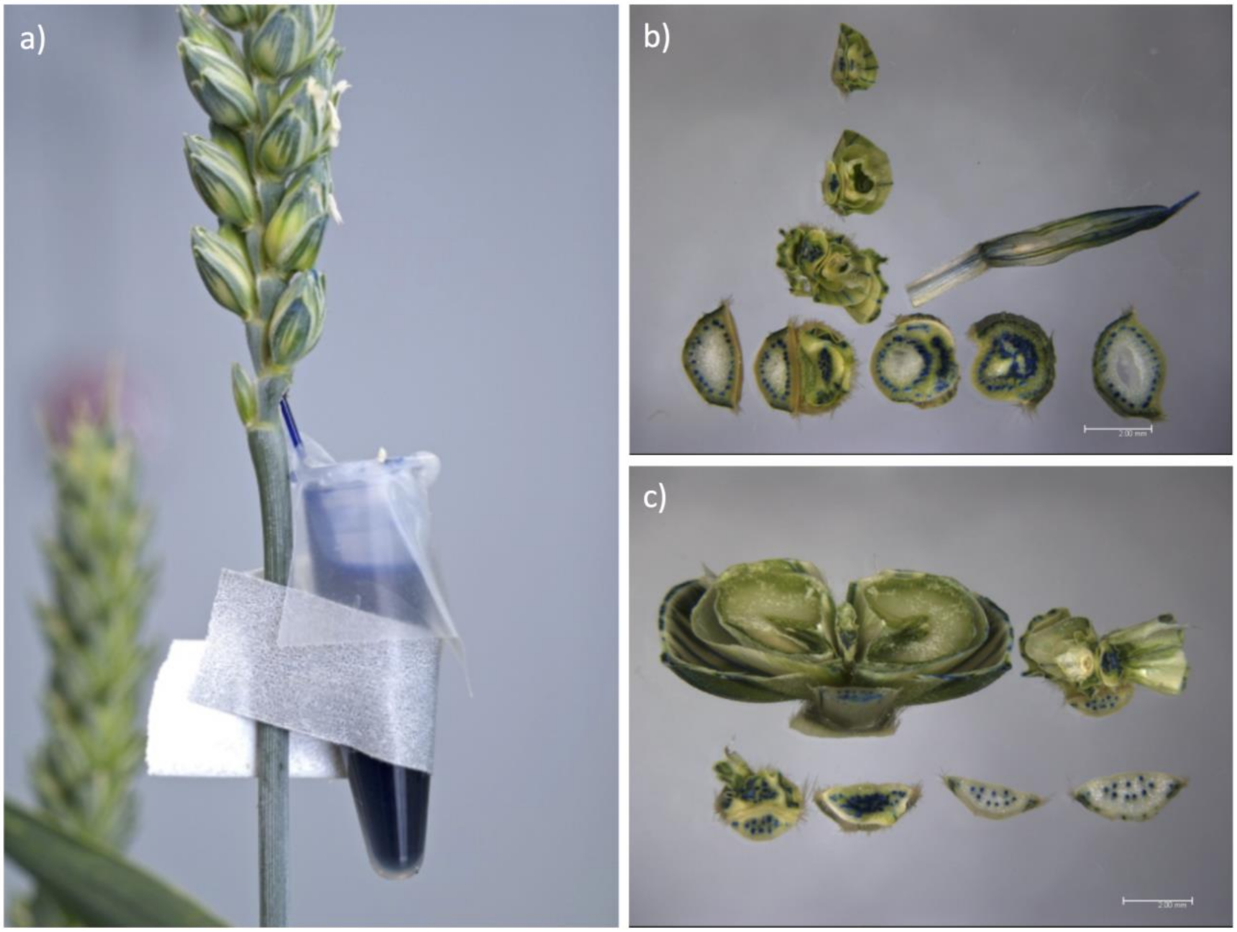

Figure. S1 Uptake of aniline blue by a wheat ear at 10 dpa via capillary tube feeding. The capillary tube (a) is inserted in the rachis, opposite the first spikelet and it is immersed in an Eppendorf containing the dye solution. The dye is evenly distributed in the vascular bundles of the rachis (b), spikelets and florets (c).

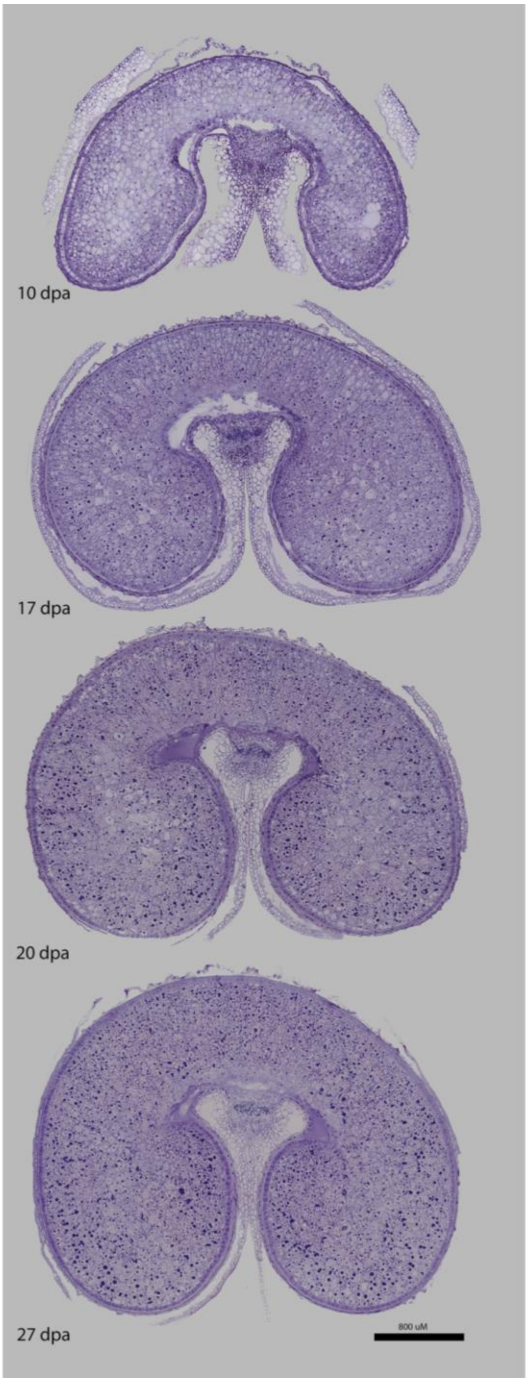

Figure. S2 Transverse sections of the developing caryopses prepared by ultramicrotomy and stained with toluidine blue to show protein distribution in the starchy endosperm at 10 dpa, 17 dpa, 20 dpa and 27 dpa.

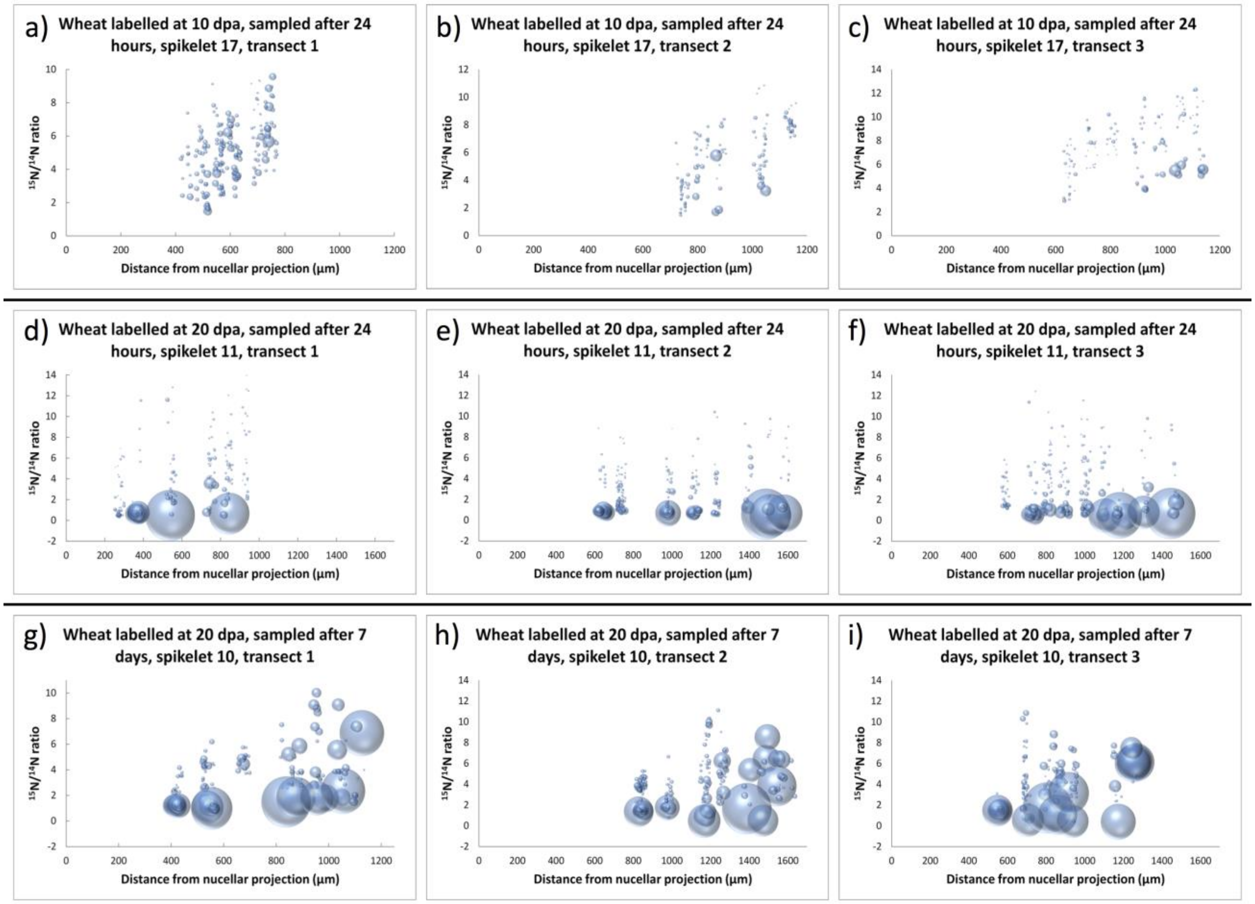

Figure. S3 A comparison of the three transects shown in Figure 1 using a graphical representation to show the size and ^{15}N enrichment of protein bodies. Graphs a-c show data from starchy endosperm tissue after labelling at 10 dpa 24 hours, graphs d-f after labelling at 20 dpa 24 hours and graphs g-i after labelling at 20 dpa 7 days. Essentially the same pattern is observed from all three transects.

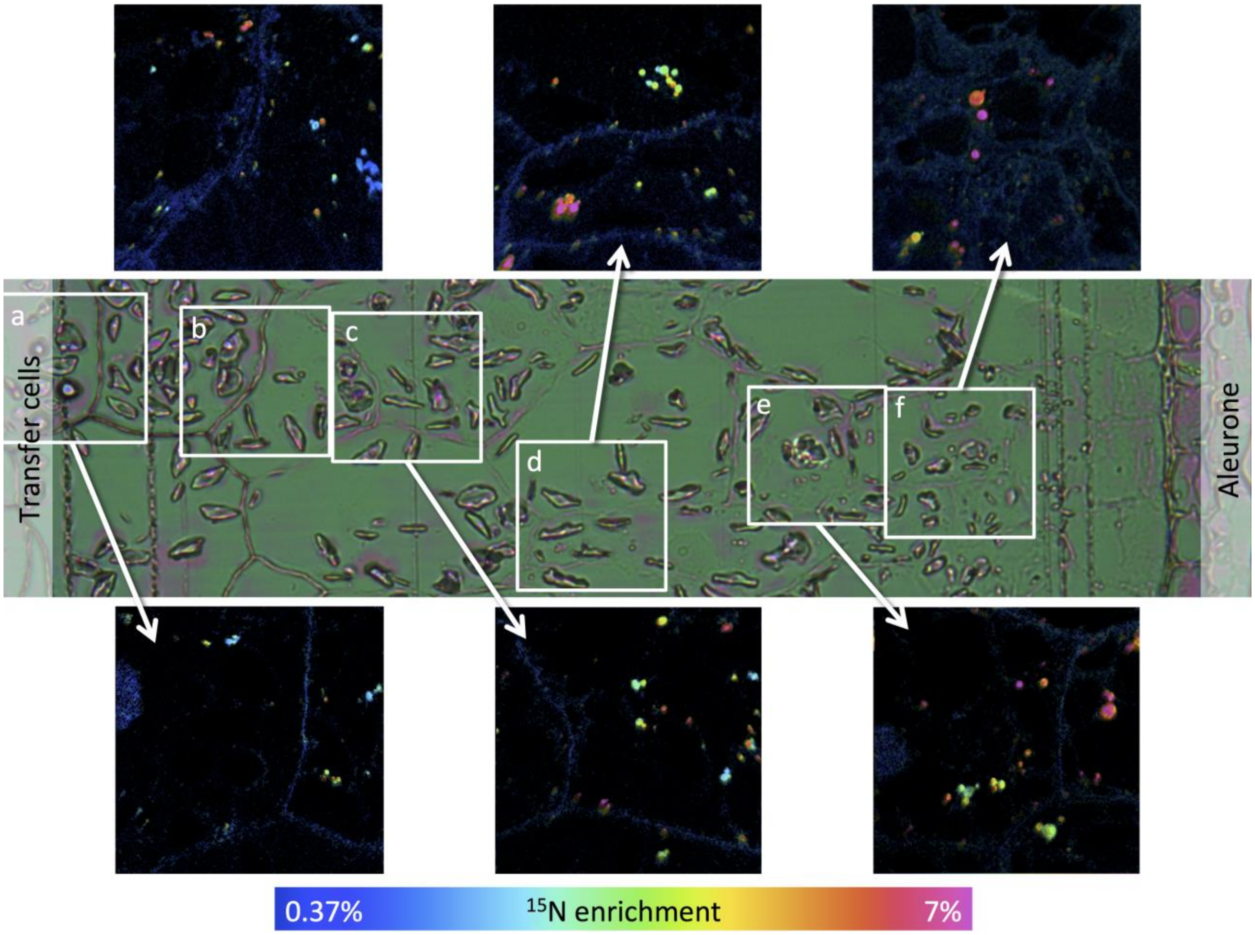

Figure. S4 Analysis of transect 1 of a wheat starchy endosperm taken from developing caryopses at 11 dpa, after feeding ^{15}N at 10 dpa. The central panel shows an optical image which was used to select the areas where secondary ion NanoSIMS images were acquired at high lateral resolution. Areas a to f marked on this optical image are expanded in the boxes and enrichment with ^{15}N is shown using a hue saturation intensity colour scale with the ^{15}N enrichment shown in the scale at the bottom.

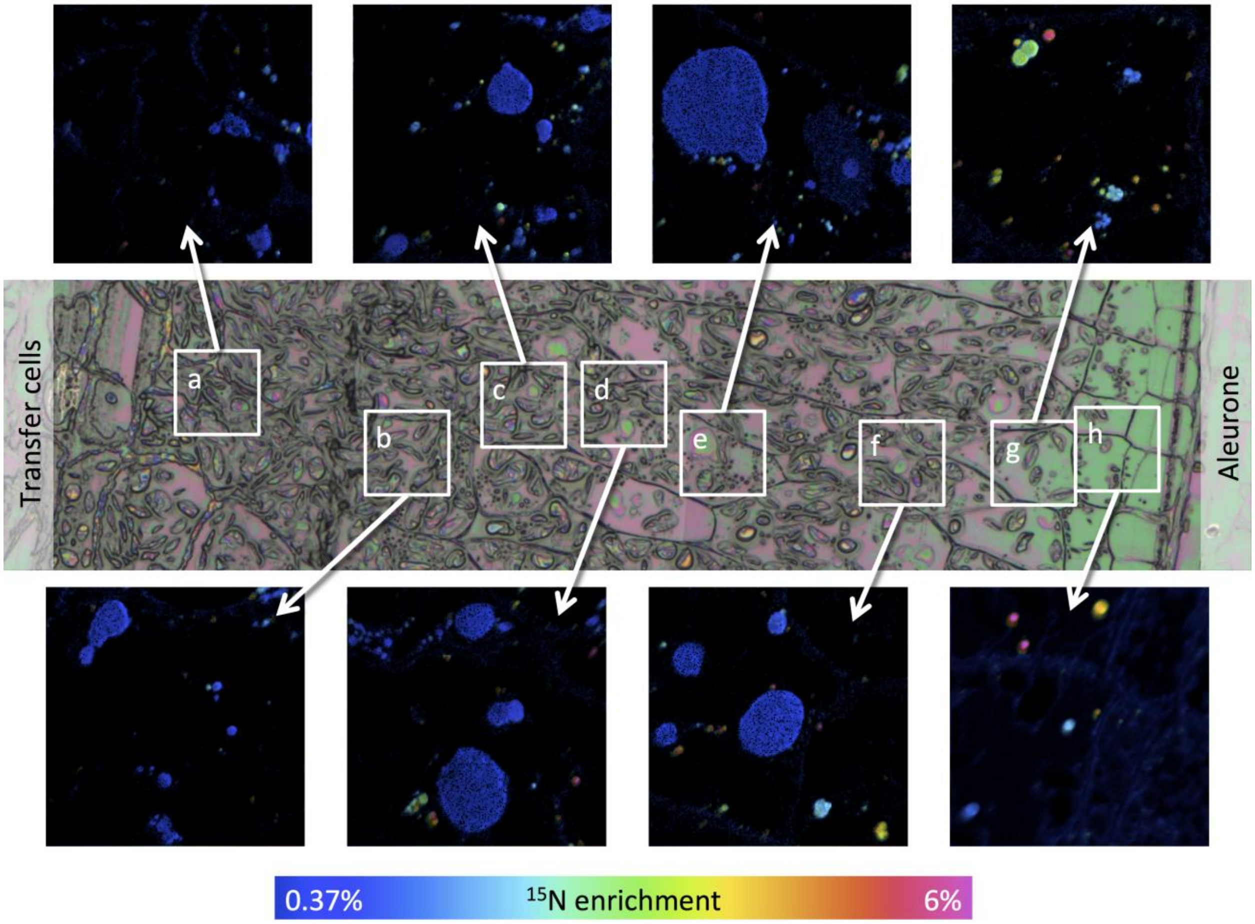

Figure. S5 Analysis of transect 1 of a wheat starchy endosperm taken from developing caryopses at 21 dpa, after feeding ^{15}N at 20 dpa. The central panel shows an optical image which was used to select the areas where secondary ion NanoSIMS images were acquired at high lateral resolution. Areas a to h marked on this optical image are expanded in the boxes and enrichment with ^{15}N is shown using a hue saturation intensity colour scale with the ^{15}N enrichment shown in the scale at the bottom.

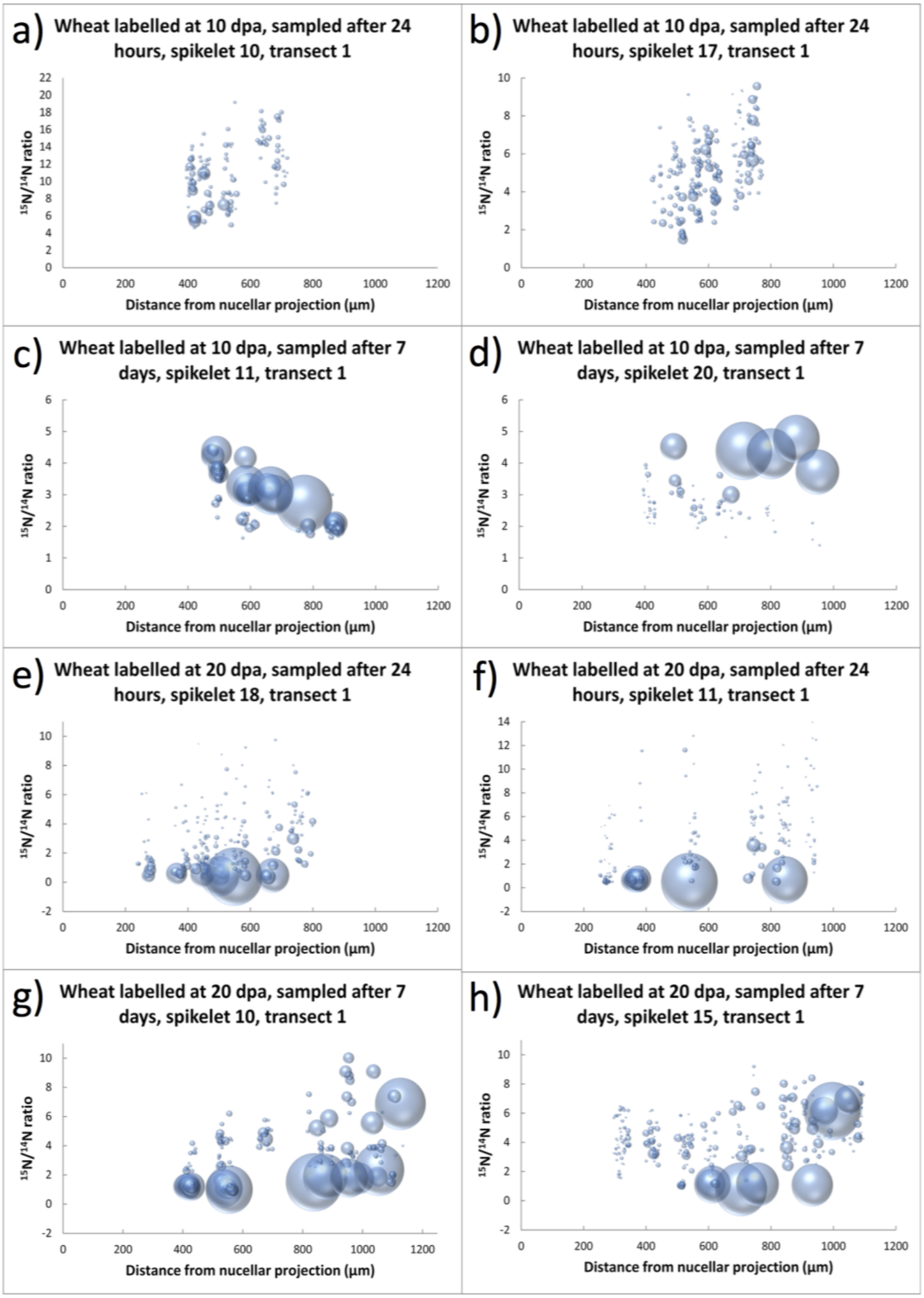

Figure. S6 Graphical representation of the size and ^{15}N enrichment of protein bodies along transect 1 of starchy endosperm tissue after labelling at 10 dpa 24 hours (a and b), 10 dpa 7

days (c and d) 20 dpa 24 hours (e and f) and 20 dpa 7 days (g and h). Adjacent graphs compare data from different spikelets and show a good level of similarity in the replicates.

Table S1 Bulk enrichment data from developing wheat caryopses at 6 hours, 24 hours and 7 days after the commencement of feeding with ^{15}N at 10 and 20 days days post anthesis (dpa).

	**Mean caryopsis dry wt (mg)**	**d ^{15} N**	**% ^{15}N**	**% N**
control	0.55	3.60	0.3676	1.69
control	0.42	14.19	0.3715	1.89
10 dpa 6 hrs	0.54	22337.71	8.5486	2.34
10 dpa 6 hrs	0.54	22484.84	8.6025	2.60
10 dpa 24 hrs	0.44	26589.92	10.1062	3.88
10 dpa 24 hrs	0.43	27013.78	10.2614	2.83
10 dpa 7 days	0.51	13876.97	5.4494	2.77
10 dpa 7 days	0.52	11525.44	4.5881	2.45
20 dpa 6 hrs	0.54	3879.03	1.7872	1.40
20 dpa 6 hrs	0.54	4147.63	1.8856	1.75
20 dpa 24 hrs	0.52	13120.93	5.1725	1.84
20 dpa 24 hrs	0.47	11814.65	4.6940	1.71
20 dpa 7 days	0.53	10504.93	4.2143	1.93
20 dpa 7 days	0.57	10205.93	4.1047	1.69
