# Supplementary material for: The dynamics of protein body formation in developing wheat grain
Source: Plant Biotechnol J. 2016 Mar 15;14(9):1876–82. doi: 10.1111/pbi.12549 (PMC4988504; doi:10.1111/pbi.12549)
Supplement: Supplementary file 2 — Supplementary Legends [file PBI-14-1876-s002.docx]

**Supporting information**

Additional supporting information may be found in the online version of this article

**Figure S1** Uptake of aniline blue by a wheat ear at 10 dpa via capillary tube feeding.

**Figure S2** Transverse sections of the developing caryopses and stained with toluidine blue.

**Figure S3** A graphical comparison of the three transects shown in Figure 1 showing the size and enrichment of the protein bodies.

**Figure S4** Analysis of transect 1 of a wheat starchy endosperm taken from developing caryopses at 11 dpa, after feeding ^15^N at 10 dpa.

**Figure S5** Analysis of transect 1 of a wheat starchy endosperm taken from developing caryopses at 21 dpa, after feeding ^15^N at 20 dpa.

**Figure S6** A comparison of the transects taken from grains labelled at 10dpa 24 hrs, 10 dpa 7 days, 20 dpa 24 hrs and 20dpa 7 days.

**Table S1** Bulk enrichment data from developing wheat caryopses at 6 hours, 24 hours and 7 days
